# Fully automated ^18^F-fluorination of *N*-succinimidyl-4-[^18^F]fluorobenzoate ([^18^F]SFB) for indirect labelling of nanobodies

**DOI:** 10.1038/s41598-022-23552-8

**Published:** 2022-11-04

**Authors:** Surasa Nagachinta, Paolo Novelli, Yoann Joyard, Nicolas Maindron, Patrick Riss, Sylvestre Dammicco

**Affiliations:** 1 GIGA-CRC In Vivo Imaging, Université de Liège, Liège, Belgium; 2 ORA Neptis, Philippeville, Belgium

**Keywords:** Biochemistry, Biomarkers, Molecular medicine

## Abstract

*N*-succinimidyl-4-[^18^F]fluorobenzoate ([^18^F]SFB), a widely used labeling agent to introduce the 4-[^18^F]fluorobenzoyl-prosthetic group, is normally obtained in three consecutive steps from [^18^F]fluoride ion. Here, we describe an efficient one-step labeling procedure of [^18^F]SFB starting from a tin precursor. This method circumvents volatile radioactive side-products and simplifies automatization. [^18^F]SFB was obtained after HPLC purification in a yield of 42 + 4% and a radiochemical purity (RCP) > 99% (n = 6). In addition, we investigate the automation of the coupling of [^18^F]SFB to a nanobody (cAbBcII10, targeting β-lactamase enzyme) and purification by size exclusion chromatography (PD-10 desalting column) to remove unconjugated reagent. Production and use of [^18^F]SFB were implemented on a radiosynthesis unit (Neptis^®^). The fully automated radiosynthesis process including purification and formulation required 160 min of synthesis time. [^18^F]SFB-labeled nanobody was obtained in a yield of 21 + 2% (activity yield 12 + 1% non-decay corrected) and a radiochemical purity (RCP) of > 95% (n = 3). This approach simplifies [^18^F]SFB synthesis to one-step, enhances the yield in comparison to the previous report and enables the production of radiolabeled nanobody on the same synthesis module.

## Introduction

Due to its physical and chemical characteristics, fluorine-18 (^18^F) is the preferred radionuclide for PET imaging. Efficient production by means of the ^18^O(p,n)^18^F reaction on [^18^O]H_2_O targets, a half-life of 109.7 min and the lowest maximum positron energy in nature result in an excellent commercial potential. The reasons to label biomolecules with ^18^F are the possibility to work with high activity, allowance to transport the production, availability of cyclotron and ease of process. Indeed, contrary to labelling methods involving a radiometal, ^18^F labelling allows to work with an unmodified protein without addition of chelating agent. Covalent labeling of small molecules through stable C−F bonds allows for broad applicability of the radionuclide in PET radiotracers. As direct labeling with [^18^F]fluoride ion involves harsh conditions such as organic solvent, high temperature and basic pH, this technique may be incompatible with fragile protein samples^1–3^. Given this concern, a number of ^18^F-labelled prosthetic groups have been developed for bioconjugation to sensitive biomolecules^4–6^.

*N*-Succinimidyl-4-[^18^F]fluorobenzoate ([^18^F]SFB), a well-known potential prosthetic agent, reacts with nucleophilic heteroatoms, for example lysine side chains and *N*-terminal primary amino groups of peptides and proteins to form corresponding [^18^F]fluorobenzamides^7,8^. It has been used in a variety of nuclear medicine applications to radiolabel proteins, peptides and antibodies^9–11^. Although radiosynthesis of [^18^F]SFB via an automated process has been described in the literature, the described processes are complicated and often involve multistep synthesis incompatible with typical single stage synthesis modules^12–14^. In addition, the associated risks of carrying the radionuclide through multiple steps involving volatile radioactive side-products ([^18^F]CH_3_F and [^18^F]fluorobenzaldehyde ([^18^F]FBA)) is problematic for the majority of PET centres^15^. Therefore, further simplification of the radiosynthesis of [^18^F]SFB benefits its widespread availability. Alternative methods to labelling with prosthetic groups are direct labelling, [^18^F]AlF or click chemistry. Several recent reviews present all these different techniques^16,17^. However, labelling with a group such as [^18^F]SFB (lysine) or [^18^F]FBEM (cysteine) represent an undeniable advantage since unmodified protein can be used without addition of a chelating agent.

In 2013, Ye and Sanford introduced the mild copper-mediated fluorination of aryl stannanes and aryl trifluoroborates^18^. Soon thereafter, the radiofluorinating variant of the reaction was published by the Scott group^19,20^.The precursors for this labeling method have been explored with aryl anions masked as boronic acid derivatives and arylstannanes^21,22^. Arylstannanes are particularly appealing precursors for radiolabeling in terms of its convenient preparative access from inexpensive starting materials, stability of Sn–C bond, and feasibility of cupration of the stannane in the labelling reaction^23–25^. Thus, the development of prosthetic groups from tin precursors via Cu-mediated nucleophilic radiofluorination is an attractive option.

Nanobodies are small recombinant antigen-binding fragments derived from camelid heavy-chain only antibodies^26^. With a molecular weight of 12–15 kDa and their compact structure, they provide several advantages such as good water-solubility and (thermo)stability, high affinity and specificity as well as low immunogenicity which serve as excellent probes for molecular imaging application. The pharmacokinetics of nanobodies are favorable compared to full-size antibodies due to the rapid accumulation to their targets after intravenous administration, while unbound molecules are quickly cleared from the bloodstream through renal elimination, rendering the high signal-to-background ratios. As a consequence, nanobodies are good candidates for imaging applications in oncology, immunology and cardiovascular disease^27–29^. For this study, we selected a universal VHH framework nanobody (cAbBcII10) targeting β-lactamase enzyme. 

**Figure 1:**
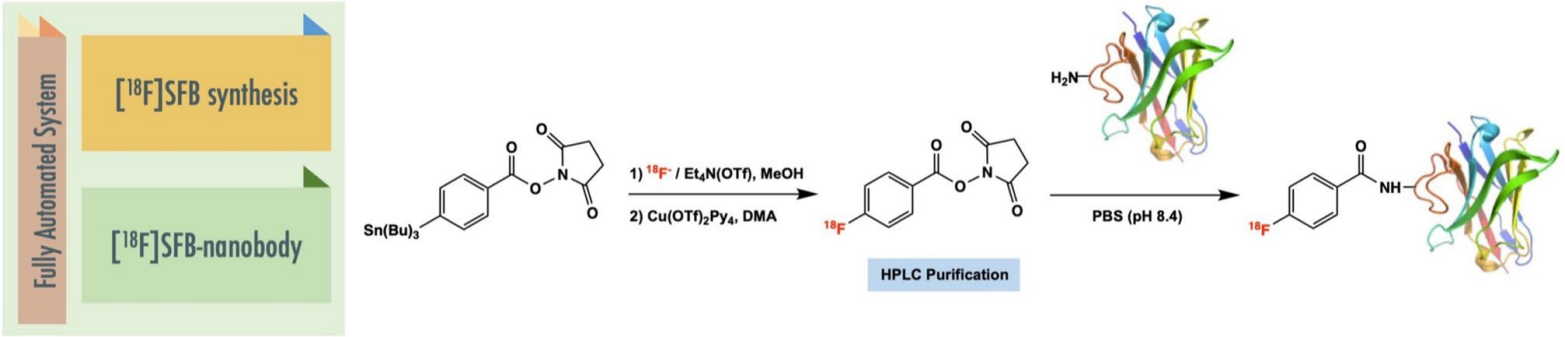
Fully automated system of [^18^F]SFB-nanobody.

**Figure 2:**
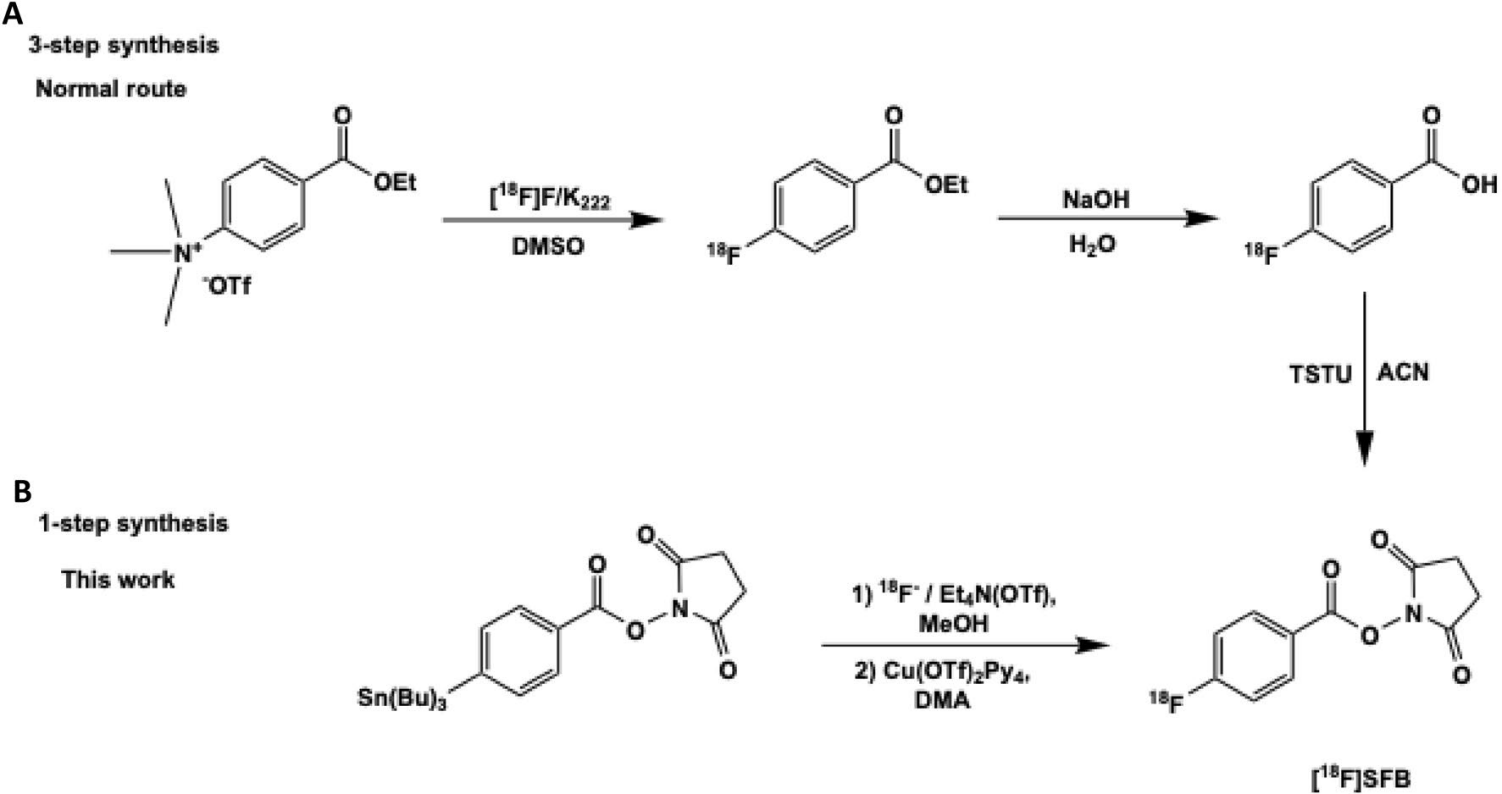
Standard 3-step procedure (**A**) and novel one-step method (**B**) of the synthesis of [^18^F]SFB.

The goal of this study was to simplify the radiosynthesis of [^18^F]SFB and perform the automated conjugation with a nanobody on a Neptis^®^ synthesizer (Fig. 1). [^18^F]SFB was synthesized in one step to reduce the complexity of its common 3-step synthesis previously reported in the literatures (Fig. 2). The copper-mediated radiofluorination is not in basic condition like in the usual [^18^F]-labeling protocol with base/K_222_. Using (Et_4_N)OTf as the eluent provided advantages to allow labeling with fluoride and prevent the hydrolysis of the active ester. Although one-step synthesis of [^18^F]SFB from spirocyclic iodonium ylide precursors has been published^30^, this is the first effort towards a one-step radiolabeling procedure to synthesize [^18^F]SFB from a stannyl precursor by Cu-mediated nucleophilic fluorination. Moreover, to avoid manual manipulation of the radioactive product for purification of the [^18^F]SFB-conjugated nanobody and proof of the value of a direct route, the coupling reaction has been automated including purification on a PD-10 desalting column installed on the module.

To our knowledge, this is the first time that the fully automated one-step radiosynthesis of [^18^F]SFB from tin precursor and the subsequent ^18^F-labeling biomolecule including purification were reported on the same synthesis module. [^18^F]SFB-nanobody was obtained with high RCP and in good yield. This synthesis allows for exploration of its potential as a prosthetic group for fully automatic radiolabeling of biomolecules for positron emission tomography (PET) tracers.

## Materials and methods

### Materials

4-iodobenzoic acid, *N,N'*-Dicyclohexylcarbodiimide (DCC), *N*-hydroxysuccinimide, tetrakis(triphenylphosphine)palladium (Pd(PPh_3_)_4_), tetrakis(pyridine)copper(II)triflate (Cu(OTf)_2_Py_4_), tetraethylammonium trifluoromethanesulfonate (N(Et)_4_(OTf)), and solvents (dimethylacetamide, dichloromethane, ethyl acetate, methanol, heptane, toluene, and acetonitrile) were obtained from Merck KGaA (Sigma Aldrich brand, Darmstadt, Germany). Hexabutylditin was supplied from alfa aesar (Haverhill, MA, USA). *N*-succinimidyl 4-(tri-*n*-butylstannyl)-benzoate and 1-{[(4-Fluorophenyl)carbonyl]oxy} pyrrolidine-2,5-dione were purchased from abcr GmbH (Karlsruhe, Germany). Oasis WCX Plus Short Cartridge (225 mg Sorbent per Cartridge, 60 µm), Oasis HLB Plus Short Cartridge (225 mg Sorbent per Cartridge, 60 µm), Sep-Pak tC18 Plus Short Cartridge (400 mg Sorbent per Cartridge, 37–55 µm) and Oasis MAX 1 cc Vac Cartridge (10 mg Sorbent per Cartridge, 30 µm) were bought from Waters (Milford, MA, USA). PD-10 desalting column was acquired from Cytiva (Marlborough, MA, USA). Thin-layer chromatography (TLC) (normal phase plates, silica gel 60 coated with fluorescent indicator F254s) was obtained from Macherey–Nagel (Duren, Germany).

### Instruments and software

Water used in all experiments was deionized from Milli-Q Integral Water Purification System. A Bioscan TLC scanner model AR2000 was used to determine the fraction of radioactivity on the TLC-plates corresponding to the product by Bio-Chrom plus software v3.3 (LabLogic Systems Ltd., Sheffield, United Kingdom). The purification of [^18^F]SFB was performed on a SymmetryPrep C18 column (10 × 150 mm; 5 μm) using an isocratic gradient (Acetonitrile/H_2_O:36/64) with a flow rate of 4 mL/min. UPLC-MS chromatography was performed on a ACQUITY H-Class sytem with TQD on a UPLC^®^BEH C18 column (2.1 × 100 mm; 1.7 μm) was analyzed with the gradient flow of 0.5 mL/min (Acetonitrile/H_2_O: 5/95–100/0 from 0 to 3 min, 100/0–5/95 from 3 to 6 min). The UPLC chromatograms were processed with Empower 3 software (Milford, MA, USA) for evaluation. The nanobody was analyzed by Biozen size exclusion columns (0.1 M Na_2_PO4 + 0.025% NaN_3_, pH 6.8, 0.3 mL/min) from Phenomenex (Torrance, CA, USA).

### Synthesis of *N*-succinimidyl 4-iodobenzoate (2)

*N*-Succinimidyl 4-iodobenzoate was synthesized from 4-iodobenzoic acid (0.50 g, 2.01 mmol), N-hydroxysuccinimide (0.255 g, 2.21 mmol) and dicyclohexyl carbodiimide (DCC, 0.59 g, 2.85 mmol) dissolved in dichloromethane (15 mL)^31^. After an overnight of stirring, the white precipitates were filtered off, and the filtrate was evaporated using a rotary evaporator. The solid residue was suspended in a 1:1 mixture of dichloromethane/hexane and filtered. The insoluble material was recrystallized from methanol to yield white crystals of *N*-Succinimidyl 4-iodobenzoate in a yield of 55% (1.11 mmol). Identity and purity of the product were confirmed by UPLC-MS (calculated: 344.95, obtained: 345.94 (M + H^+^)) and ^1^H-NMR (CDCl_3_): δ 7.9 (d, *J* = 8.4 Hz, 2H), 7.83 (d, *J* = 8.4 Hz, 2H), 2.9 (s, 4H). Melting point is 138.1–139.3 °C and Rf of 0.55 in TLC (AcOEt/Hexanes 4:6).

### Synthesis of *N*-succinimidyl 4-(tri-*n*-butylstannyl)-benzoate (3)

*N*-Succinimidyl 4-iodobenzoate (0.89 g, 2.60 mmol), Pd(PPh_3_)_4_ (0.1 g, 0.11 mmol) and hexabutylditin (0.35 mL, 9.24 mmol) were dissolved in anhydrous toluene (50 mL) under a nitrogen atmosphere^32^. The mixture was heated under reflux until the solution turned black (24 h) after which the contents of the flask were concentrated to give an oily crude mixture. The residue was purified by silica gel chromatography using dichloromethane as eluent to yield *N*-succinimidyl 4-(tri-*n*-butylstannyl)-benzoate (67%, 1.74 mmol). For confirmation of the purity and identity of the product after purification the isolated material was analyzed by UPLC-MS (calculated: 508.24, obtained: 527.25 (M + H_3_O^+^)) and ^1^H-NMR (CDCl_3_): δ 8.039 (d, *J* = 8.4 Hz, 2H), 7.62 (d, *J* = 8 Hz, 2H), 2.91 (s, 4H), 0.89–1.55 (m, 27H). Rf of 0.8 in TLC (AcOEt/Hexanes 4:6).

### Synthesis of *N*-benzoyloxysuccinimide (5)

*N*-hydroxysuccinimide (1.47 g, 12.50 mmol, 1.25 equiv.) was added to a solution of benzoic acid (1.22 g, 10.0 mmol, 1.0 equiv.) and *N,N'*-dicyclohexylcarbodiimide (DCC, 2.27 g, 11.0 mmol, 1.10 equiv.) in ethyl acetate (40 mL) at room temperature^33^. After 2 h, 60 mL of ethyl acetate was added into the suspension and the mixture was kept at -80 °C for 2 h. It was filtrated to remove the sideproduct (dicyclohexylurea, DCU) precipitate and washed with cold ethyl acetate. The filtrate was concentrated under reduced pressure. White crystal of *N*-benzoyloxysuccinimide was isolated by crystallization in EtOAc-EtOH (1.91 g, 8.71 mmol, 87%). The product was determined by UPLC-MS and ^1^H-NMR (CDCl_3_): δ = 8.13 (m, 2H, *H*_ar-*meta*_), 7.68 (m, 1H, *H*_ar-*para*_), 7.51 (m, 2H, *H*_ar-*ortho*_), 2.90 (s, 4H, C*H*_2_). Melting point is 137.1–138.2 °C and Rf of 0.55 in TLC (AcOEt/Hexanes 4:6). 

### Radiolabeling of [^18^F]SFB

A compact kit-less synthesizer developed by Neptis^®^ (Fig. S1A,B) was used for the automated production of fluorine-18 radiotracers. Production of 2.1 ± 0.2 GBq (n = 6) [^18^F]fluoride ion was achieved via the ^18^O(p,n) ^18^F reaction (cyclotron, IBA, Belgium) by irradiation of an isotopically enriched [^18^O]H_2_O target. The activity was trapped on the Oasis MAX Cartridge and eluted into the reactor with 10 mg (0.04 mmol) of N(Et)_4_(OTf) in 600 μL of MeOH. The eluate was concentrated at 90 °C under a stream of nitrogen for 12 min. Then, 0.7 mL of *N*-succinimidyl 4-(tri-*n*-butylstannyl)-benzoate (11 mg, 0.02 mmol) and Cu(OTf)_2_Py_4_ (30 mg, 0.04 mmol) in anhydrous DMA was added to the reactor. After labeling for 10 min at 140 °C, the reaction mixture was diluted with water and the mixture was passed through Oasis WCX and HLB SPE cartridges in series. The cartridges were rinsed with 13% acetonitrile in water to remove impurities. Crude [^18^F]SFB was eluted from the HLB cartridge with 2 mL acetonitrile and checked for metal residues by ICP-MS. Then it was diluted with 2 mL water and purified with semi-preparative HPLC in order to isolate [^18^F]SFB from by-products that may interfere with the labeling of the biomolecule. The collected fraction of the purified [^18^F]SFB with a retention time of 12–14 min was subjected to QC with UPLC. To study molar activity, the activity was retrapped on tC18 and eluted with CH_3_CN It was calculated by assaying radioactivity and its associated mass determined by the area under UV peak at 240 nm against a standard mass curve.

### Automation of [^18^F]SFB labeled nanobody

The fraction of purified [^18^F]SFB from HPLC was collected in a vial containing 60 mL of water. The solution was passed through a Sep-Pak tC18 Plus Short Cartridge and, afterwards, the product was eluted with diethyl ether (1 mL) into the reactor. Finally, the mixture was evaporated to dryness under a stream of nitrogen at 40 °C for 10 min and cooled down for 5 min. Nanobody dissolved in 0.3 mL PBS buffer (cAbBCII10, 1.25 and 3.75 mg/mL, pH 8.4) was added to the dried [^18^F]SFB in the reactor and incubated for 30 min at room temperature. The final purification of [^18^F]SFB-nanobody was conducted on a PD-10 desalting column implemented on the module. The fraction of purified [^18^F]SFB-nanobody (3 mL) was eluted with PBS buffer (pH 8.2) and analyzed with size exclusion chromatography (SEC). The radiochemical purity (RCP) was also determined by acetonitrile precipitation and thin layer chromatography (TLC). In case of acetonitrile precipitation, the final solution containing (n = 3) the radiolabeled nanobody was incubated for 5 min in a 1/1 mixture with acetonitrile. Next, the samples were centrifuged, after which the protein-associated radioactivity in the resulting pellet was measured.

## Results and discussion

### Synthesis of the stannyl precursor

**Figure 3:**
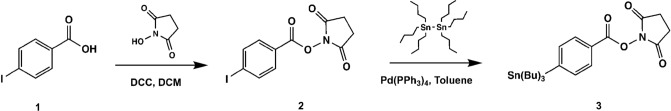
Synthesis of the stannyl precursor.

The synthesis of the stannyl precursor from 4-iodobenzoic acid (compound 1) consists of two steps (Fig. 3). *N*-Succinimidyl 4-iodobenzoate (compound 2) and *N*-succinimidyl 4-(tri-*n*-butylstannyl)-benzoate (compound 3) were successfully prepared in good yields (55% and 67% respectively). The purities were found to be greater than 99% as characterized by analytical UPLC-MS and ^1^H-NMR.

### Radiolabeling of [^18^F]SFB

**Figure 4:**
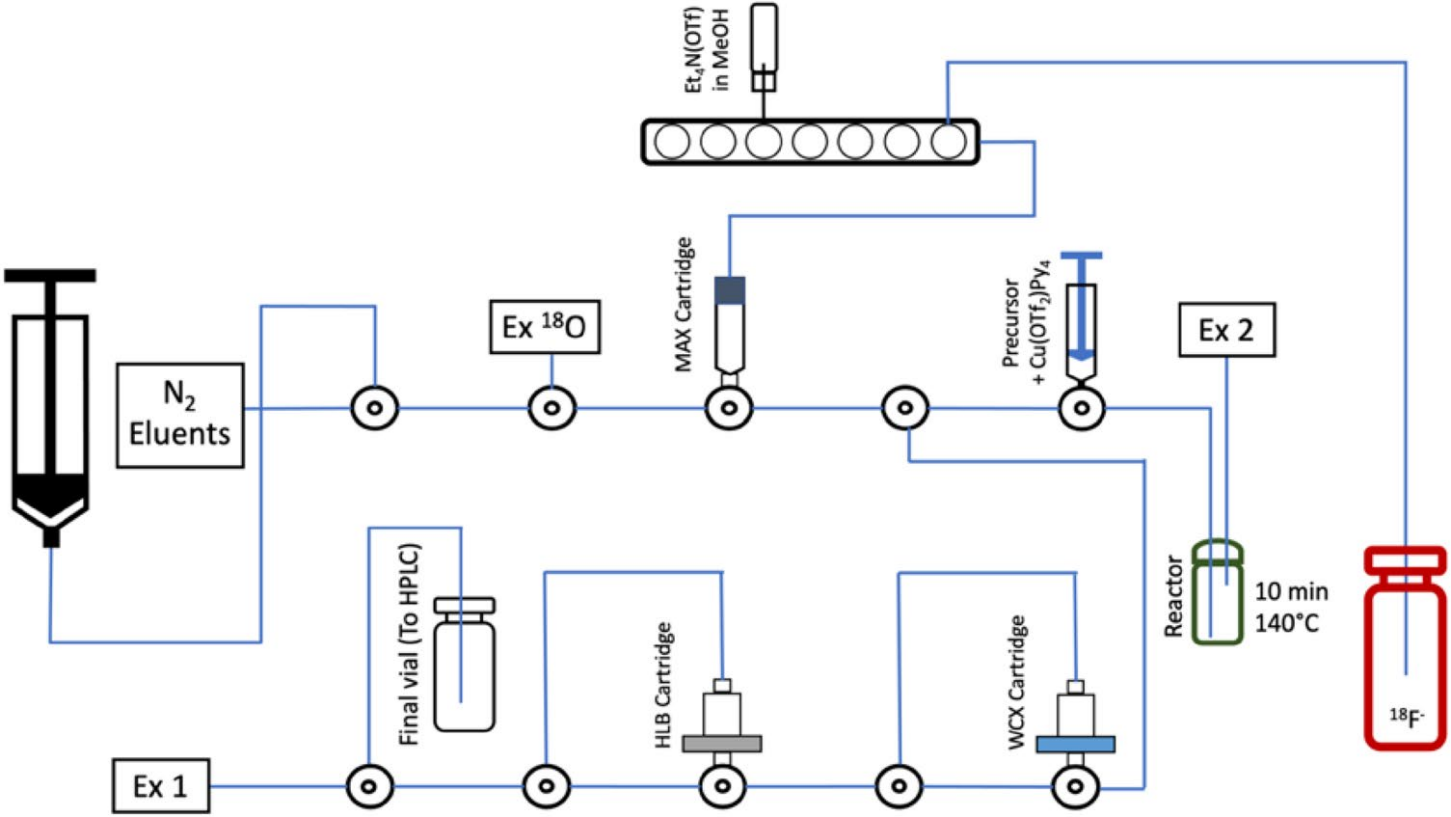
Layout of the module for the synthesis of [^18^F]SFB.

[^18^F]SFB (compound 4) has been synthesized with a novel one-step method from a tributyltin precursor (*N*-succinimidyl 4-(tri-*n*-butylstannyl)-benzoate) by Cu-mediated nucleophilic fluorination. The radiosynthesis procedure and layout were shown in Figs. 2 and 4. The crude reaction mixture was transferred through an Oasis WCX and HLB cartridge connected in series and rinsed with 13% acetonitrile to remove impurities. As residual Cu species were trapped on the WCX cartridge, the [^18^F]SFB was obtained in a metal-free form via elution from the HLB cartridge. The yield of [^18^F]SFB was 44 + 4% (decay corrected) with a radiochemical purity (RCP) > 99% (n = 20). This one-step labeling reduced the complexity of the synthesis and avoided the formation of volatile side-products^12–15^. Residual amounts of Cu and Sn in the final formulated solution from the synthesis of [^18^F]SFB (n = 12) as determined by ICP-MS were found to be lower than the daily exposure limits specified in the ICH Guidelines^34^ (0.002 + 0.001 and 0.084 + 0.046 μg vs. 340 and 640 μg/day, respectively).

**Figure 5:**
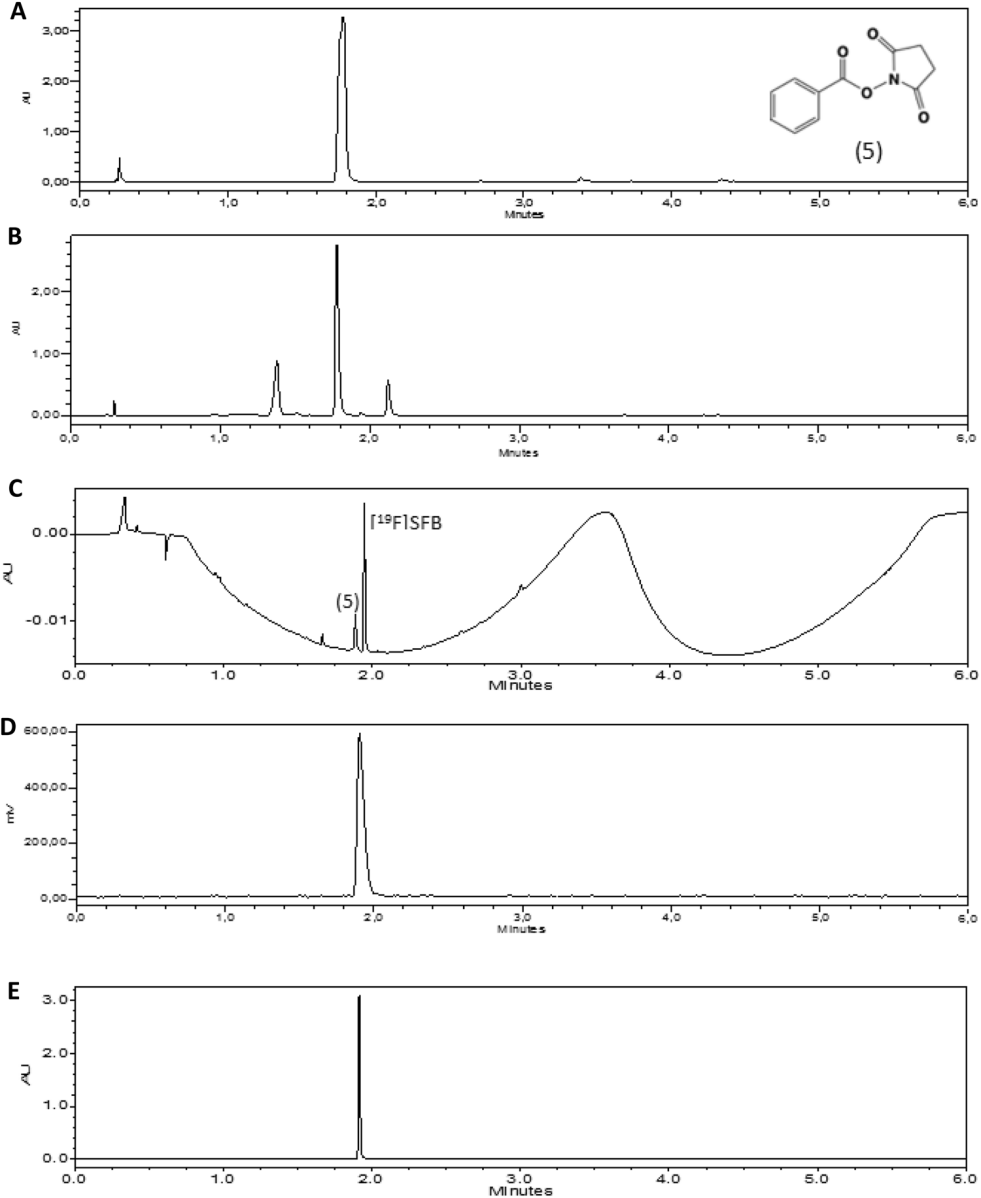
UPLC chromatogram (UV detector, 240 nm) (**A**) N-benzoyloxysuccinimide, (**B**) crude [^18^F]SFB, (**C**) purified [^18^F]SFB, (**D**) UPLC chromatogram (γ detector) of purified [^18^F]SFB and (**E**) [^19^F]SFB.

Unfortunately, coupling nanobody with [^18^F]SFB from the synthesis of the module without HPLC purification was not successful. This is due to the nonradioactive impurity from protodestannylation reaction of the precursor that was able to conjugate with the biomolecules and thus decreased the “apparent” specific activity. To confirm our hypothesis about the impurity, *N*-benzoyloxysuccinimide (5) standard was prepared and analyzed in UPLC (Fig. 5A). Its peak was detected in the crude [^18^F]SFB obtained from the module (the second peak, retention time at 1.8 min) (Fig. 5B). According to its calibration curve, we had *N*-benzoyloxysuccinimide 12 + 1% (n = 3) converted from tin precursor after the synthesis of [^18^F]SFB (Fig. S2A). Therefore, HPLC purification before the conjugation of the nanobody was required to eliminate the proto-destannylation of the precursor. The purified [^18^F]SFB fraction from semipreparative HPLC offered 42 + 4% yield with RCP > 99% (n = 6) (Fig. 5C,D) and corresponded to [^19^F]SFB reference (Fig. 5E). This yield is higher than the previous one-step synthesis starting from spirocyclic iodonium ylide precursors, which provided 3–22% of [^18^F]SFB^30^. Molar activity of [^18^F]SFB was 17 GBq/μmol at the end of the synthesis calculated from [^19^F]SFB standard curve (Fig. S2B).

### Automation of [^18^F]SFB labeled nanobody

**Figure 6:**
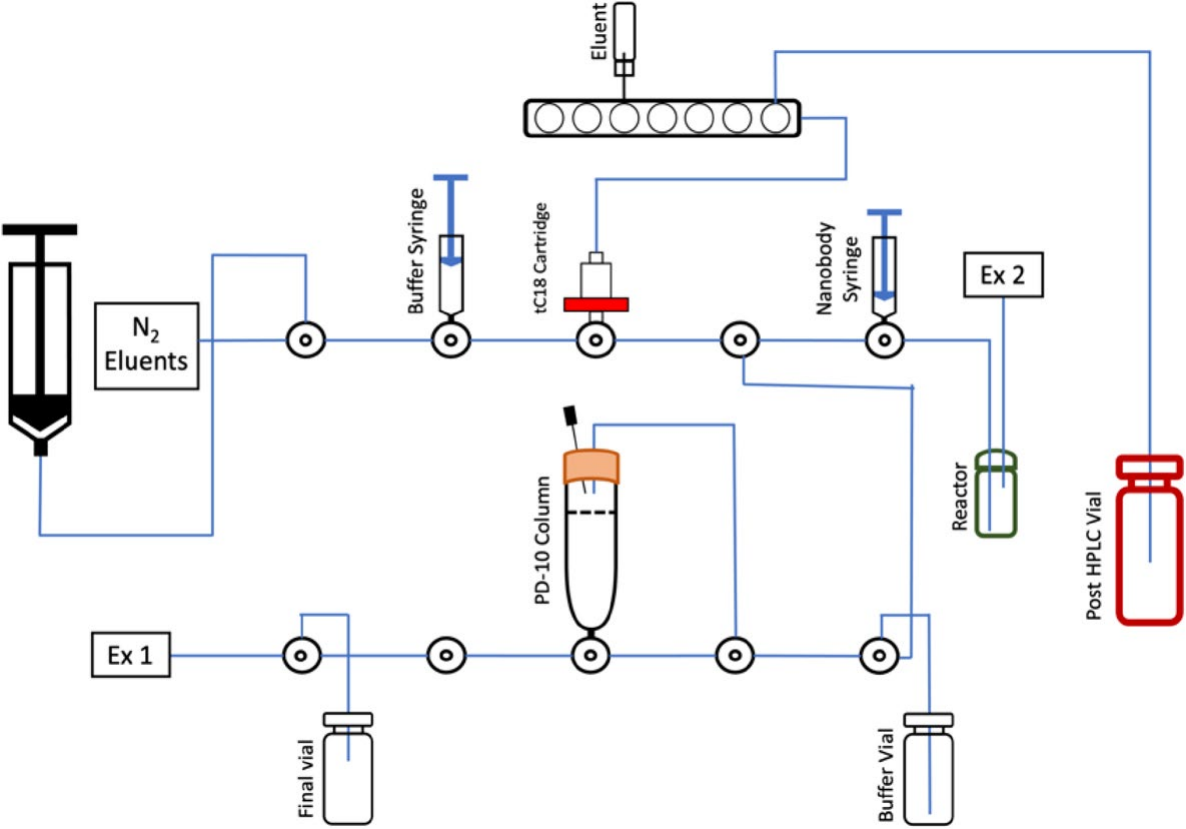
Layout of the module for [^18^F]SFB labeled nanobody and purification.

**Figure 7:**
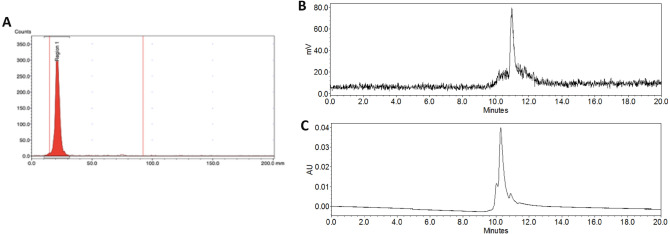
Purified [^18^F]SFB-nanobody (**A**) Radio-TLC, (**B**) size-exclusion chromatography (γ detector) and (**C**) reference nanobody (UV detector, 280 nm).

[^18^F]SFB labeled nanobody and the PD-10 purification were automatically performed on the module (Fig. 6). The purified [^18^F]SFB obtained from HPLC was trapped on tC18 and eluted with diethyl ether to facilitate the evaporation in the reactor. After the drying step, 0.3 mL of nanobody (pH 8.4) was added from the syringe to the reactor and labeled for 30 min at room temperature. This specific pH was selected because a pH below 8.5 lowered the reactivity the amino groups toward acylation, while a pH above 8.5 induced the degradation of [^18^F]SFB^35^. The reaction mixture was diluted with 1 mL of PBS buffer (pH 8.2) from the syringe, transferred to the 10 mL syringe (black) and pushed on top of the PD-10 column. We mimicked the gravity purification by creating the vacuum by slowly pulling up the 10 mL syringe. The first 1.5 mL fraction was discarded since it had no product. Then, PBS buffer from the buffer vial was added on top of the column. The purified fraction of [^18^F]SFB conjugated nanobody was collected in the final vial (3 mL) in a yield of 5 + 1% (n = 4) and 21 + 2% (n = 3) for nanobody at 1.25 and 3.75 mg/mL respectively. The higher yield of [^18^F]SFB conjugated nanobody in the higher concentration nanobody resulted from the more lysine residues that increased the conjugation. Moreover, in the higher concentration nanobody, there was less nanobody lost during the purification step on the PD-10 column, which might be caused by nonspecific binding. The product had a radiochemical purity of more than 95% (Fig. 7A). These data were confirmed by acetonitrile precipitation with more than 95% of the activity remaining in the protein-associated resulting pellet. SEC confirmed that purified [^18^F]SFB labeled nanobody corresponded to its reference nanobody (Fig. 7B,C). The small difference in retention time between reference nanobody and [^18^F]SFB labeled nanobody originated from the delay of the UV and gamma detectors caused by the low flow of the eluent (0.3 mL/min).

With a significant reduction in its overall complexity, this automated and concise synthesis would make the routine production of [^18^F]SFB more practical and attractive for ^18^F-labeled biomolecules, enabling research tools to enhance clinical studies.

## Conclusions

A one-step labeling procedure for [^18^F]SFB was developed on the Neptis^®^ synthesizer. As compared to the usually applied 3‐step synthesis, this method reduced the complexity of the procedure, shortened the synthesis time, and avoided the formation of volatile radioactive side-products. This synthesis of [^18^F]SFB afforded reproducible yield with high radiochemical purity and showed chemically pure in which the residual metal impurities were lower than the daily limit for humans. Furthermore, the conjugation of [^18^F]SFB with nanobody and its purification was firstly automatically performed on the same module. This fully automated bioconjugation resulted in a reproducible labeling yield and minimized exposure of the production personnel to the radioactivity.

## Supplementary Information

Supplementary Figures.

## Data Availability

All data generated or analyzed during this study are included in this published article [and its supplementary information files].
