# Supplementary material for: Fully automated 18F-fluorination of N-succinimidyl-4-[18F]fluorobenzoate ([18F]SFB) for indirect labelling of nanobodies
Source: Sci Rep. 2022 Nov 4;12:18655. doi: 10.1038/s41598-022-23552-8 (PMC9636270; doi:10.1038/s41598-022-23552-8)
Supplement: Supplementary file 1 — Supplementary Figures. [file 41598_2022_23552_MOESM1_ESM.pdf]

Supplementary material

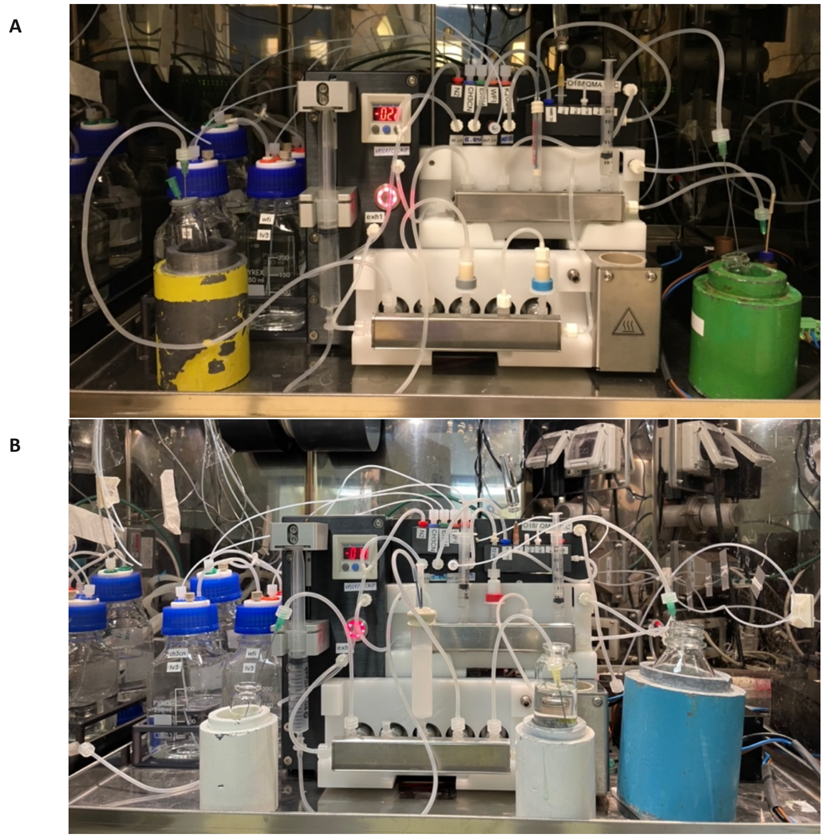

Figure S1. Radiosynthesizer for the synthesis of (**A**) [^{18}F]SFB and (**B**) [^{18}F]SFB-nanobody.

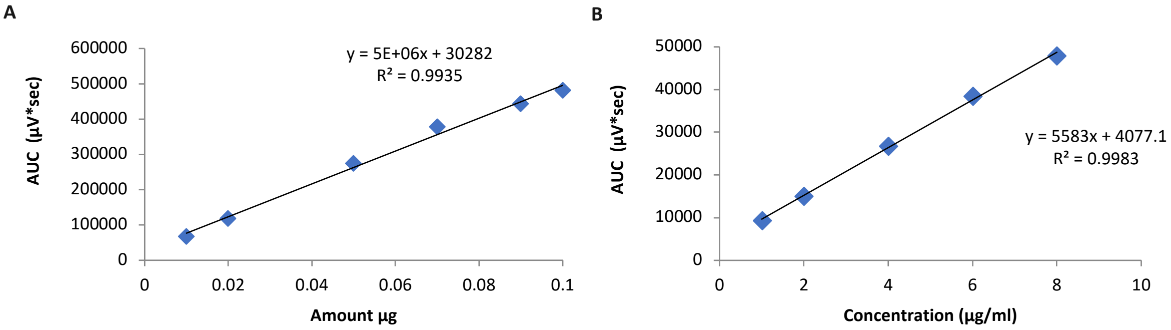

Figure S2. Standard calibration curve of (**A**) (5) and (**B**) [^{19}F]SFB.
